# Advances in bioengineered CAR T/NK cell therapy for glioblastoma: Overcoming immunosuppression and nanotechnology‐based strategies for enhanced CAR T/NK cell therapy

**DOI:** 10.1002/btm2.10716

**Published:** 2024-08-31

**Authors:** Nasim Dana, Arezou Dabiri, Majed Bahri Najafi, Azadeh Rahimi, Sayed Mohammad Matin Ishaghi, Laleh Shariati, Minmin Shao, Assunta Borzacchiello, Ilnaz Rahimmanesh, Pooyan Makvandi

**Affiliations:** ^1^ Applied Physiology Research Center, Cardiovascular Research Institute Isfahan University of Medical Sciences Isfahan Iran; ^2^ Department of Microbiology, Faculty of biological science and technology The University of Isfahan Isfahan Iran; ^3^ Department of Biomaterials, Nanotechnology and Tissue Engineering, School of Advanced Technologies in Medicine Isfahan University of Medical Sciences Isfahan Iran; ^4^ Biosensor Research Center, School of Advanced Technologies in Medicine Isfahan University of Medical Sciences Isfahan Iran; ^5^ Department of Otorhinolaryngology, The Dingli Clinical College affiliated to Wenzhou Medical University Wenzhou Central Hospital Wenzhou 325000 China; ^6^ Institute of Polymers, Composites and Biomaterials National Research Council (IPCB‐CNR) Naples Italy; ^7^ The Quzhou Affiliated Hospital of Wenzhou Medical University Quzhou People's Hospital Zhejiang China; ^8^ Centre of Research Impact and Outreach Chitkara University Rajpura 140417 Punjab India; ^9^ University Centre for Research & Development Chandigarh University Mohali Punjab India

**Keywords:** cell therapy, chimeric antigen receptors, glioblastoma multiforme, nanotechnology, tumor microenvironment

## Abstract

Glioblastoma is a strong challenge in the worldwide field of central nervous system malignancies. GBM's inherent heterogeneity, along with the formation of an immunosuppressive tumor microenvironment, supports its resistance to current therapy methods. Immunotherapeutic methods have emerged as potential options in recent years. However, because of the inherent limits of traditional immunotherapeutic techniques innovative approaches are required. Advances in cut‐edge techniques provide a possible route for improving effector cell effectiveness. This review gives insight into the complicated immunosuppressive pathways in GBM, with a particular emphasis on CAR T/NK‐cell treatment as a potential achievement. Recognizing and addressing these concerns might open the way for more effective and focused glioblastoma therapies, providing hope for the future with the aim of improved outcomes for patients. In addition, this review presents valuable insights into the integration of nanotechnology into CAR T/NK cell therapy for enhanced efficiency of these personalized gene therapy products.

**Abbreviations**
APCs: antigen‐presenting cells
BBB: blood‐brain barrier
CAFs: cancer‐associated fibroblasts
CAIX: carbonic anhydrase IX
CAR: chimeric‐antigen receptor
CCL2: C–C motif chemokine ligand 2
CCR2: C–C motif chemokine receptor 2
CLTX: chlorotoxin
CLTX‐CAR T: chlorotoxin‐directed CAR T
CMV‐specific: cytomegalovirus‐specific
CRS: cytokine release syndrome
CSF‐1: colony‐stimulating factor 1
CSF‐1R: colony‐stimulating factor 1 receptor
CSR: chimeric switch receptor
CTLA‐4: cytotoxic T‐lymphocyte‐associated protein 4
CYP: cytochrome P450
DC: dendritic cells
ECM: extracellular matrix
ECs: endothelial cells
EGF: epidermal growth factor
FAP‐α: fibroblast activation protein
FDA: Food and Drug Administration
FGF: fibroblast growth factor
GAMs: glioma‐associated resident microglia and peripheral‐invading monocyte‐derived macrophages
GBM: glioblastoma
GSK3: glycogen synthase kinase 3
GVHD: graft‐versus‐host disease
HA: hyaluronic acid
HAase: hyaluronidase
HAS: hyaluronic acid synthases
HER2: human epidermal growth factor receptor 2
HIFs: hypoxia‐inducible factors
HiTA system: hypoxia‐inducible transcription amplification system
HPSE: heparanase
HRE: hypoxia response element
ICAM‐1: intercellular adhesion molecule‐1
ICB: immune checkpoint blockade
ICs: immune checkpoint molecules
IFN‐γ: interferon‐γ
IL‐10: interleukin 10
IL‐13Ra2: interleukin‐13a2
IL‐6: interleukin 6
ILT2: Ig‐like transcript 2
IR: ionizing radiation
iTregs: induced Tregs
KIR: killer cell immunoglobulin‐like receptors
KIR2DL4: killer cell immunoglobulin‐like receptor, two Ig domains, and long cytoplasmic tail 4
mAbs: monoclonal antibodies
MDMs: monocyte‐derived macrophages
MDSCs: myeloid‐derived suppressor cells
MGMT: O6‐methylguanine DNA methyltransferase
MHC: major histocompatibility complex
NK Cell: natural killer cell
NKG2D: natural killer group 2, member D
NKG2D: natural‐killer group 2, member D
nTregs: natural Tregs
PD‐1: programmed death 1
PDGF‐R: platelet‐derived growth factor receptor
PDL1: programmed death‐ligand 1
PHD2: prolyl‐4‐hydroxylase 2
PP2A: protein phosphatase 2a
ROI: region of interest
scFv: single‐chain variable fragment
SIRPα: signal‐regulatory protein Α
TAAs: tumor‐associated antigens
TAMs: tumor‐associated macrophages
TAMs: tumor‐linked macrophages
TCR: T‐cell receptor
TEAM: T‐cell engaging antibody molecule
TEM: tumor endothelial marker
TGF‐β: transforming growth factor beta
TKIs: tyrosine kinase inhibitors
TME: tumor microenvironment
TNBC: triple‐negative breast cancer
Treg: regulatory T cells
VCAM‐1: vascular cell adhesion molecule‐1
VEGF: vascular endothelial growth factor
VEGFR2: vascular endothelial growth factor receptor 2
VH: antibody heavy chains
VL: antibody light chains
wtEGFR: wild‐type EGFR

## 1 INTRODUCTION

GBM is one of the most malignant and invasive central nervous system tumors, accounting for nearly 13 million deaths worldwide in 2022.^1^, ^2^ Surgical resections followed by concurrent radiation and chemotherapy are currently the gold standard of care for GBM treatment. However, GBM is still an incurable disease; hence, new strategies are needed to treat it successfully.^3^ The inherent heterogeneity of this tumor type and the development of an immunosuppressive tumor microenvironment are two potential underlying mechanisms responsible for this shortcoming.^3^

Recently, immunotherapies have held considerable potential for the treatment of cancer by retraining and utilizing the patient's immune response against malignancies. These techniques are increasingly being employed to treat a variety of cancers, including brain tumors.^4^ Several immunotherapy methods are used to suppress tumors, including mAbs, adoptive cell therapy, therapeutic vaccines, and CAR T/NK cells.^5^, ^6^, ^7^, ^8^, ^9^, ^10^ Peptide or dendritic cell have proven to be extremely precise and secure, however, have not yet demonstrated their effectiveness in treating cancer in medical settings. This is intended for various factors, such as tumor diversity, self‐acceptance, and immune system repression.^11^ Effector cells, such as T cells and NK cells, can potentially expand ex vivo, followed by administration to patients. The latest developments in gene editing have made it feasible to enhance the capabilities of these effector cells. These enhancements encompass a range of biological functions, such as generating cytokines, identifying multiple antigens, and augmenting cell trafficking proficiency. Gene editing offers a promising avenue to mitigate the suppressive immune conditions observed in glioblastoma by inhibiting immune‐inhibitory molecules. As a result, there is an improvement in the effectiveness of effector cells in terms of their cytotoxicity, endurance, and overall safety. Exciting possibilities for readily available immunotherapy solutions against glioblastoma involve utilizing allogeneic CAR T cells. These cells are engineered to minimize the risks of GVHD and rejection reactions. Another avenue consists of using induced pluripotent stem cell‐derived NK cells that carry CARs with signaling domains specifically tailored for NK cell functioning.^12^, ^13^

Although there have been encouraging outcomes in lymphomas and diffuse intrinsic pontine gliomas, initial findings in GBM have not shown any therapeutic benefits. The limited number of specific antigens in GBM, the heterogeneous expression of TAA, and the antigen loss after starting antigen‐specific therapy due to immune‐editing could be potential explanations for this phenomenon.^14^ Notably, since GBM exhibits severe local and systemic immunosuppression, the efficacy of such therapeutic strategies is restricted. Indeed, improving patient survival with immunotherapy is severely hampered by this overt immunosuppression. Most immunotherapies appear destined for failure without addressing this immunosuppression in GBM.^15^ Several well‐known clinical trials of immunotherapies in GBM have been unsuccessful in establishing a therapeutic advantage. In light of this, a better conception of the shortcomings of present therapies could lead to more effective treatment in the future. In addition, the prevalence of redundant tumor‐mediated immune suppression systems is a significant obstacle to the application of immune therapy in the treatment of glioblastoma. Integrating new advancements in CAR‐T engineering procedures may enhance CAR‐T‐cell effectiveness against glioblastoma, prolong the presence of CAR‐T cells within the body, and reduce off‐target and on‐target off‐tumor and specific side effects on healthy tissues.^16^, ^17^

Here, we will discuss these pathways of immunosuppression, focusing on CAR T/NK cell therapy as a potential treatment for glioblastoma. This is followed by reviewing clinical trials in this field. To this aim, the glioblastoma immunosuppressive tumor microenvironment will be highlighted along with a discussion on chimeric antigen receptor T/NK cells' structure and function. The potential of CAR T/NK cells in targeting glioblastoma immunosuppressive tumor microenvironment will be discussed. Subsequently, the nanotechnology‐based approaches to overcome certain CAR T/NK cell restrictions are discussed. Finally, the role of NPs in boosting CARs functions are summarized. Subsequently, the nanotechnology‐based approaches to overcome certain CAR T/NK cell restrictions are discussed. Finally, the role of NPs in boosting CARs functions are summarized.

## 2 GLIOBLASTOMA IMMUNOSUPPRESSIVE TUMOR MICROENVIRONMENT

The microenvironment within glioma tumors, often referred to as the TME, is composed of a combination of tumor cells and diverse non‐cancerous cell types. These nonmalignant cells encompass nervous cells, stem cells, fibroblasts, as well as vascular and immune cells. The interplay between glioma cells and the TME influences multiple molecular pathways, supporting cancer growth, progression, and treatment resistance.^18^ Therefore, a better understanding of these interactions in TME could be helpful for more efficient therapy. In TME, immune‐related cells, including TAMs, MDSCs, T lymphocytes, NK cells, DC, and so forth, can play an anti‐tumor effect or, at the same time their interaction with glioma cells, promote the immune escape of cancer cells.^19^, ^20^

**FIGURE 1:**
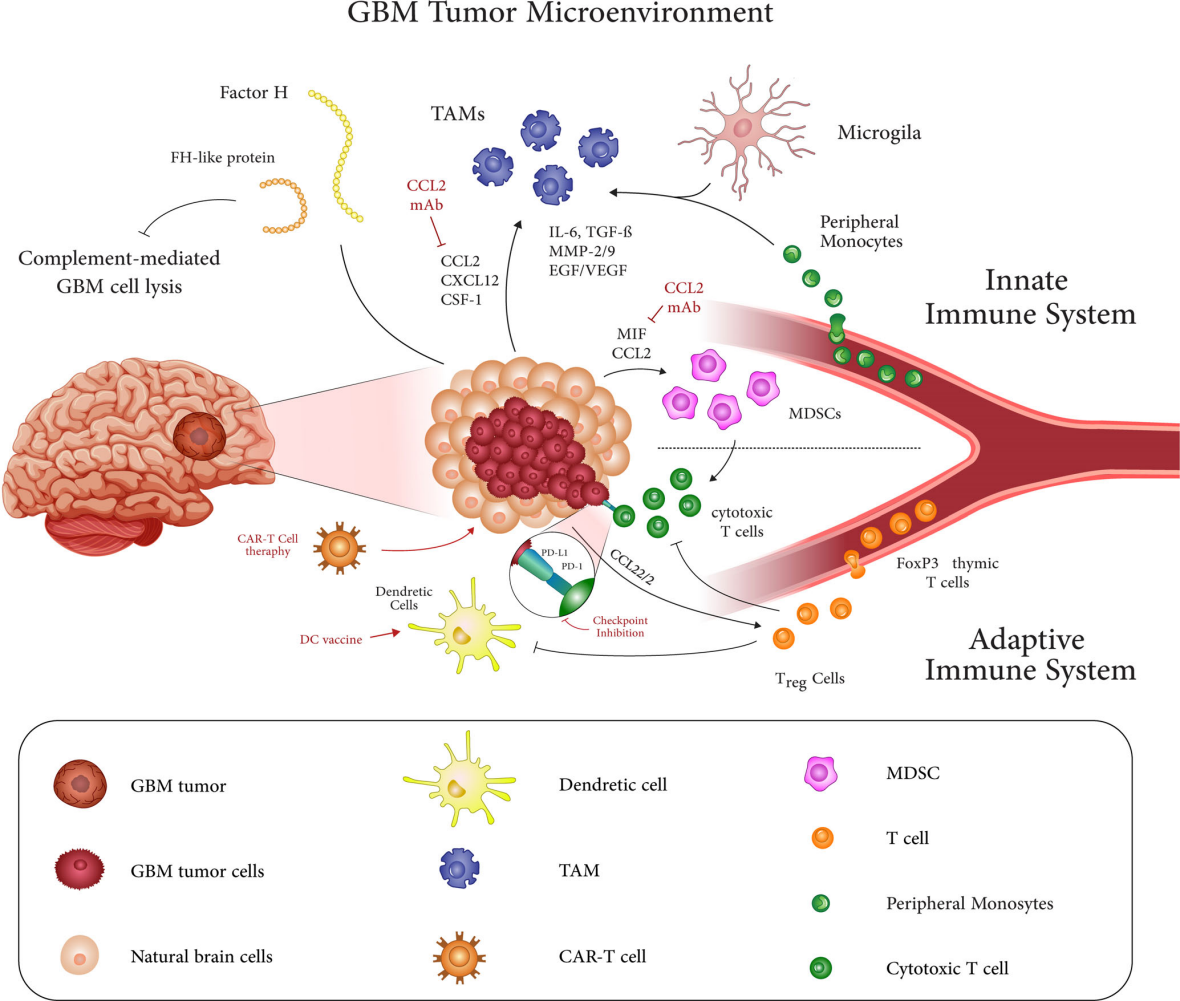
Summary of glioma‐immune cells interactions in the GBM microenvironment. Immune cells make up a significant portion of the GBM tumor microenvironment. Glioma cells secrete metabolites that alter immune cells' function and stimulate the infiltration of immunosuppressive Tregs and MDSCs into the TME, thereby changing the tumor recognition process. In addition, overexpression of metabolic enzymes inhibits antitumor responses by reducing the recruitment of NK cells and cytolytic T cells and boosting the release of inflammatory cells that attract chemokines.

Immune cells comprise a significant portion of the GBM tumor bulk, up to 30% or 40% of the total mass.^21^ The immune cell content of each tumor, which refers to the precise composition of different immune cell types within tumors, varies with the tumor stage and is associated with clinical outcomes.^4^ The brain's immune system has various physiological roles, including foreign‐substance phagocytosis, cellular debris elimination, tissue and neural regeneration, and regulation of synaptic plasticity.^22^ Glioblastoma tumors contain many immune cell types; however, immunosuppressive cells predominate^23^ (Figure 1).

Under physiological conditions, Microglia (the brain's resident macrophages) and dendritic cells are the most common immune cells in the brain. The BBB prevents immune cells and mediators from penetrating the brain and controls cellular and molecular exchange between the blood vessels and parenchyma of the brain.^24^ In pathological conditions, the invasive behavior of glioma cells breaks down the BBB, leading to vascular degeneration and increased BBB permeability to immune cells.^25^ Glioma cells secrete various chemokines, cytokines, and growth factors through a disrupted blood–brain barrier that triggers peripheral immune cell differentiation, expansion, and recruitment.^26^

Glioma cells secrete metabolites that alter immune cells' function and stimulate the infiltration of immunosuppressive Tregs and MDSCs into the TME, thereby limiting the tumor recognition process.^27^ In addition, overexpression of metabolic enzymes inhibits antitumor responses by reducing the recruitment of natural killer cells and cytolytic T cells and boosting the release of inflammatory cells that attract chemokines.^18^, ^24^

GAMs constitute the most multifunctional cell groups within the glioma TME, accounting for about 30 percent of the tumor tissue volume.^28^ During the early stages of GBM formation, microglia primarily make up the GAMs population. As GBM progresses, the number of invading macrophages/monocytes increases in response to molecular signals released by GBM, weakening the BBB's ability to attract peripheral immune cells.^29^, ^30^ GBM cells instruct GAMs to adopt an anti‐inflammatory/pro‐tumoral phenotype, leading to the production of various soluble chemicals with pro‐tumoral effects^31^, ^32^, ^33^ and affecting the GBM microenvironment, tumor development, and angiogenesis.^34^

The number of GAMs invading the tumor is linked to its grade and prognosis.^35^ GAMs are highly flexible and can be polarized into several phenotypes depending on the surroundings.^36^ GAMs can alter the immune system's response and diminish its effect on cancer cells, and by causing immune cells to take on a pro‐ or anti‐inflammatory phenotype, improve glioma cell survival. GAMs alter the structure of the ECM and make tumor cells vulnerable to invasion.^37^, ^38^ Immunoregulatory factors, such as chemokines, angiogenic and invasive factors, that stimulate the change of GAMs into the M2 type, are among the various cytokines seen in TME.^20^ The active interface between glioma cells and their TME is critical for tumor growth and evolution but poses significant therapeutic hurdles.^39^

TME has an impact on the phenotypic differentiation of GAMs. M1 and M2 are two phenotypes of activated GAMs. Although M1 cells have anticancer effects, M2 cells have immunosuppressive capacities and help develop and proliferate glioblastoma cells.^40^ Furthermore, it has been proposed that in response to glioma cell‐secreted chemicals (such as macrophage colony‐stimulating factor), macrophages polarize towards the M2 phenotype, as seen by increased expression of CD163 and CD204.^41^ GAMs are activated in brain tumors by the release of IL‐10 and TGF‐β, transforming them into the M2 phenotype with tumor‐promoting effects.^36^ M2 macrophages produce arginase‐1, IL‐10, and TGF‐1, which inhibit T‐cell antitumor activity.^41^

On the other hand, M1 macrophage markers, such as CD74 and F11R, correlate positively with the survival of glioma patients.^42^, ^43^ Studies have shown that in a 3D in vitro model, the M2 phenotype prompts endothelial cell proliferation and angiogenesis through interactions with integrin (αvβ3) receptors and the Src‐PI3K‐YAP signaling pathway. This model effectively replicates the in vivo conditions of immunosuppression and angiogenesis observed in GBM.^44^ Other investigators have argued that resting M0 macrophages accumulate in GBM tumors^44^ or that the M1/M2 ratio of tumor‐associated macrophage indicators is associated with glioma progression.^45^ Most TAMs in GBM are predominant in the M2 phenotype, which can affect the production of several molecules and stimulate cancer cell immune evasion, invasion, explosion, and angiogenesis.^41^, ^46^, ^47^, ^48^, ^49^, ^50^ TAMs and their progenitors are important components of the TME, accounting for most of the inflammatory infiltration in the myeloid line. They account for up to 30% of the tumoral bulk, far outnumbering intra‐tumoral lymphocytes.^51^ TAMs originate from two distinct cell sources: microglia, resident cells of brain tissue derived from yolk sac progenitors, and macrophages drawn from the bloodstream as monocytes. This recruitment is prompted by chemoattractant chemicals released by tumor cells.^52^

**FIGURE 2:**
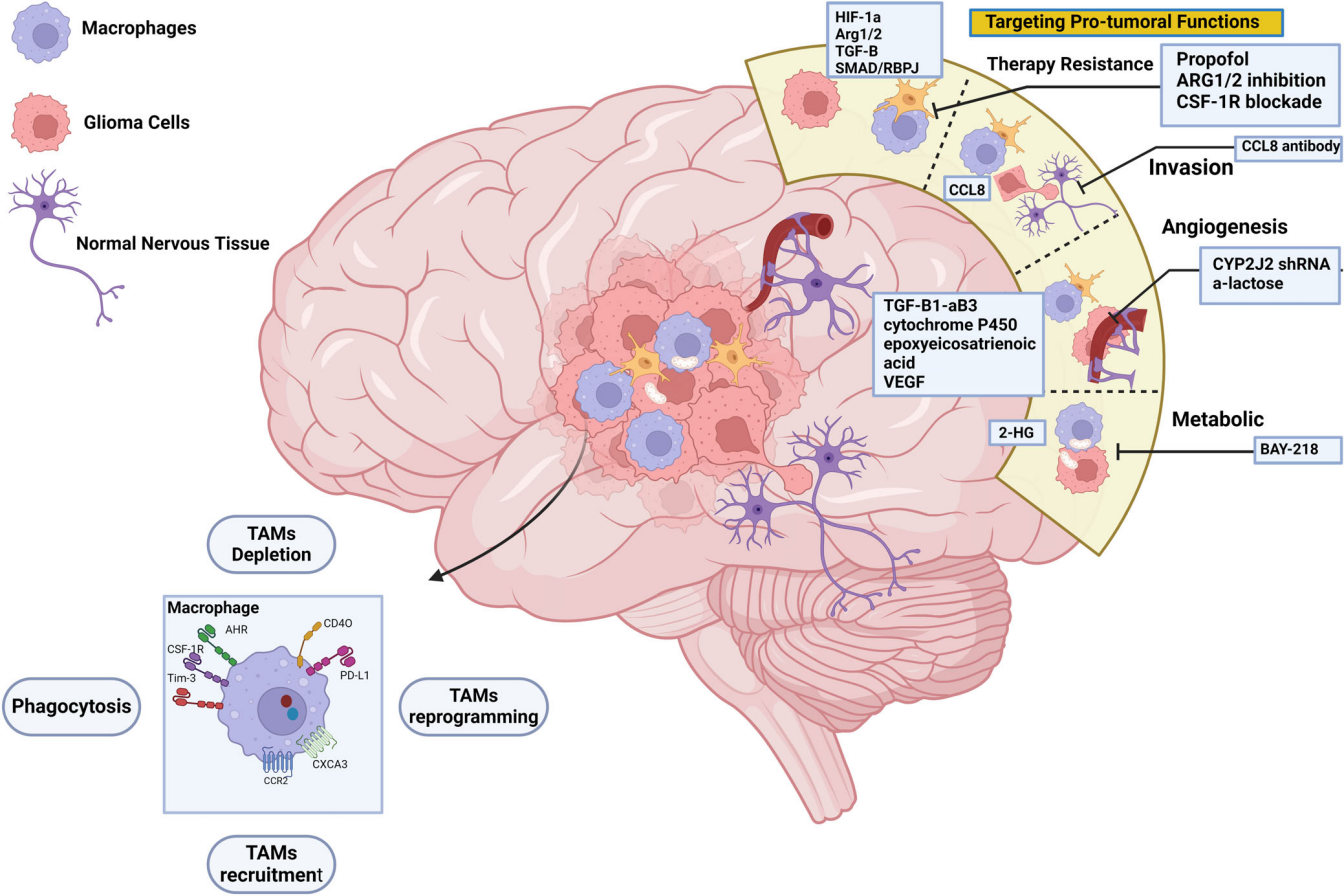
Strategies for targeting TAMs in cancer therapy. Enhancing TAMs' ability to phagocytosis tumor cells by targeting the CD47‐SIRPα axis, depleting M2‐like TAMs with drugs, preventing TAMs recruitment to cancer cells by targeting the CSF‐1‐CSF‐1R and CCL2‐CCR2 axes, and converting TAMs into M1‐like cells.

Given the link between the pro‐inflammatory milieu and the immunosuppressive environment in GBM, there is accumulating evidence indicating the development of mixed types of TAMs.^53^ TAMs stimulate GBM development and invasion through various chemicals, including stress‐inducible protein 1, EGF, TGF‐β, and IL‐6.^54^ Microglia increase tumor cell motility via the PDGF‐R.^55^ TAMs' involvement in angiogenesis is another factor contributing to the progression of GBM, and studies reported some strategies to target TAMs^56^, ^57^ (Figure 2).

In conclusion, TAMs maintain critical stages in the development of GBM by mediating communication between tumor cells and other TME components. The development of innovative therapeutics and new advancements in risk classification for GBM patients may be made possible by ongoing research.^18^

Lymphoid lineage cells play a role in TAM‐mediated immunosuppression within the glioma TME. The impact of T cells on this mechanism has been extensively examined.^58^ TAMs have been observed to impede the pro‐inflammatory response mediated by T cells at different phases of T‐cell functioning. Initially, TAMs obstruct T cells' infiltration into the GBM tissue.^59^ Intriguingly, this particular inhibition seems to affect Th1 cells more significantly than Tregs. The ability of GBMs to cause T lymphocyte inefficiency is significant, and high‐grade gliomas may include a lymphocytic infiltration that contains CD4+, CD8+, and CD4 T regulatory cells (CD4+ CD25+ Foxp3+).^60^ From the beginning of the tumor growth, these T cells are re‐educated in the immunosuppressive milieu of gliomas.^61^ Immune evasion of glioma is mostly due to its immunosuppressive characteristics.^62^ Approximately 5% to 10% of human blood CD4+ T cells are Tregs, important immune system modulators and effective in preventing GVHD and autoimmune diseases by reducing tissue inflammation.^63^ Tregs are a physiologic subset of CD4+ T cells that can suppress various immune cells and mediate peripheral tolerance.^64^ Two variations of Tregs exist nTregs, originating in the thymus, and iTregs, generated when FoxP3 is activated in conventional CD4+ T cells that encounter an immunosuppressive environment.^65^ There is a significant association between glioma‐induced immunosuppression and Tregs.^62^ CD4+ FoxP3+ Tregs increase in developing gliomas and have an active phenotype.^66^

Regulatory T cells possess the capability to release IL‐10 and TGF‐β, substances that aid in quelling the activities of other immune cells. Additionally, they possess the capacity to prompt recruited CD4+ T cells within the TME to transition into novel Tregs, termed adaptive Tregs. While Tregs are not commonly found in regular human brain tissue, a substantial contingent of immunosuppressive Tregs is discernible within the glioma microenvironment. Their infiltration into the tumor aligns with the grade of the tumor.^67^ As a result, the GBM microenvironment is strongly immunologically suppressed by the small population of Tregs.^68^

The prevalence of NK cells varies with the glioma subtype. They form the least frequent population among immune infiltrating cells in the GBM tumor microenvironment (constituting approximately 2% marked by CD3 and CD56+).^69^ When glioma patients are compared to unaffected controls, less NK is observed in the blood.^70^ NK cells release cytokines, including IFN‐γ and TNF‐α, and are well recognized for their antiviral and anti‐tumor responses.^71^

The capacity of NK cells to destroy GBM cells when triggered by IL‐2,^72^ as well as their efficiency in preventing metastasis in a GBM xenograft mouse model,^73^ have all been demonstrated in preclinical models of GBM.^72^ These cells can directly lyse the infected or cancerous cells. Similar to numerous viruses and malignancies, they utilize an adaptive mechanism to evade T‐cell detection by focusing on cells that lack MHC Class I.^74^

NK cells modulate their response to specific challenges by expressing both inhibitory and stimulatory receptors.^75^ As an example, KIR can identify MHC Class I molecules present in healthy cells, which results in the suppression of NK cell activation. On the contrary, when cells are under stress or infected, the levels of ligands that bind to the activating receptor NKG2D rise, leading to the activation of NK cells and subsequent destruction of the targeted cell.^75^

The importance of NK cells in the immune response against cancer is underscored by research demonstrating that both mice and humans with deficiencies in NK cells exhibit heightened vulnerability to specific types of cancers.^76^, ^77^

Some patient populations diagnosed with GBM exhibit diminished levels of NKG2D on the surface of their NK cells. This reduction results in a lowered activation of NK cells.^78^ Furthermore, gliomas contain a suppressive molecule called HLA‐G. This molecule can attach to specific members of the KIR family of NK receptors, including KIR2DL4 and ILT2. This attachment hampers various NK cell functions, such as cytotoxicity, secretion of IFN‐γ, activation of NKG2D, and chemotaxis.^71^ Although NK cells constitute a small proportion of tumor‐infiltrating cells, those present in the GBM TME possess characteristics that make them highly effective against tumor cells in other malignancies.^79^

The immune system's typical function involves inhibiting the proliferation of tumor cells. Studies have shown that in animal cancer models and brain cultures with organized tissue structures, microglia can impede the spread and expansion of gliomas. Nevertheless, changes in the structure of microglia and macrophages, including reorganization of their cytoskeleton, can result in the contrary outcome.^47^, ^80^, ^81^ The development of innovative therapeutics and new advancements in risk classification for GBM patients may both be made possible by ongoing research in this field.

Patients with glioblastoma are known to have severe local immunosuppression, which is a critical obstacle to overcome in immunotherapy.^15^ Several investigations have demonstrated that the immunosuppressive condition is coordinated by a variety of elements originating from gliomas, both in their membrane‐bound and soluble forms, in conjunction with the unique microenvironment within the brain.^82^ Despite the established safety and efficacy of immunotherapy in treating numerous types of cancer, its specific impact on glioblastoma remains undetermined. Management approaches for GBM patients involve combined standard treatment methods that collaborate to successfully eradicate the tumor.^83^ Moreover, the glioblastoma microenvironment acts as a barrier to anti‐tumor immune responses, so it is essential to consider this complexity when developing immunotherapies. New therapeutic approaches are desperately needed, given the poor prognosis for GBM patients receiving currently approved therapies.^82^, ^84^, ^85^ Due to extensive investments over decades in genetic and epigenetic profiling of this disease, along with studying interactions within the brain microenvironment and the immune system, there is a swift emergence of new clinical trials. Encouraging outcomes from these trials are pointing towards the potential of immunotherapy, which encompasses immune checkpoint blockade and chimeric antigen receptor T/NK cell therapy, to enhance outcomes for GBM. Ongoing studies are exploring combination therapies to reduce significant adverse effects and enhance antitumor immune responses. Therefore, we explain the rationale for CAR T/NK cells' effectiveness in the treatment of GBM, address the challenges and prospects for glioblastoma targeting using these genetically manipulated cells, and provide an update on preclinical and clinical trials in this field.

## 3 CHIMERIC ANTIGEN RECEPTOR T/NK CELLS STRUCTURE AND FUNCTION

CAR‐T‐cell treatment is an innovative and personalized cancer‐targeting strategy that has demonstrated effective and long‐lasting therapeutic outcomes. CARs, designed primarily to redirect lymphocytes, particularly T cells, are intended to identify and remove cells that display a designated target antigen.^86^ The genetically modifying autologous or allogeneic T cells to express CARs endows cells with supraphysiologic characteristics, transforming them into living therapeutic platforms. The CAR attachment to target antigens expressed on the surface of cells occurs independently of the MHC receptor, in contrast to TCRs, which directly depend on MHC for antigen presentation and are consequently restricted to specific human leukocyte antigen (HLA) expression patterns.^87^ Despite consideration of MHC or HLA expression, the T‐cell becomes activated after the CAR attaches to the tumor antigen, proliferating, resulting in robust T‐cell activation and potent anti‐tumor responses.^88^, ^89^

**FIGURE 3:**
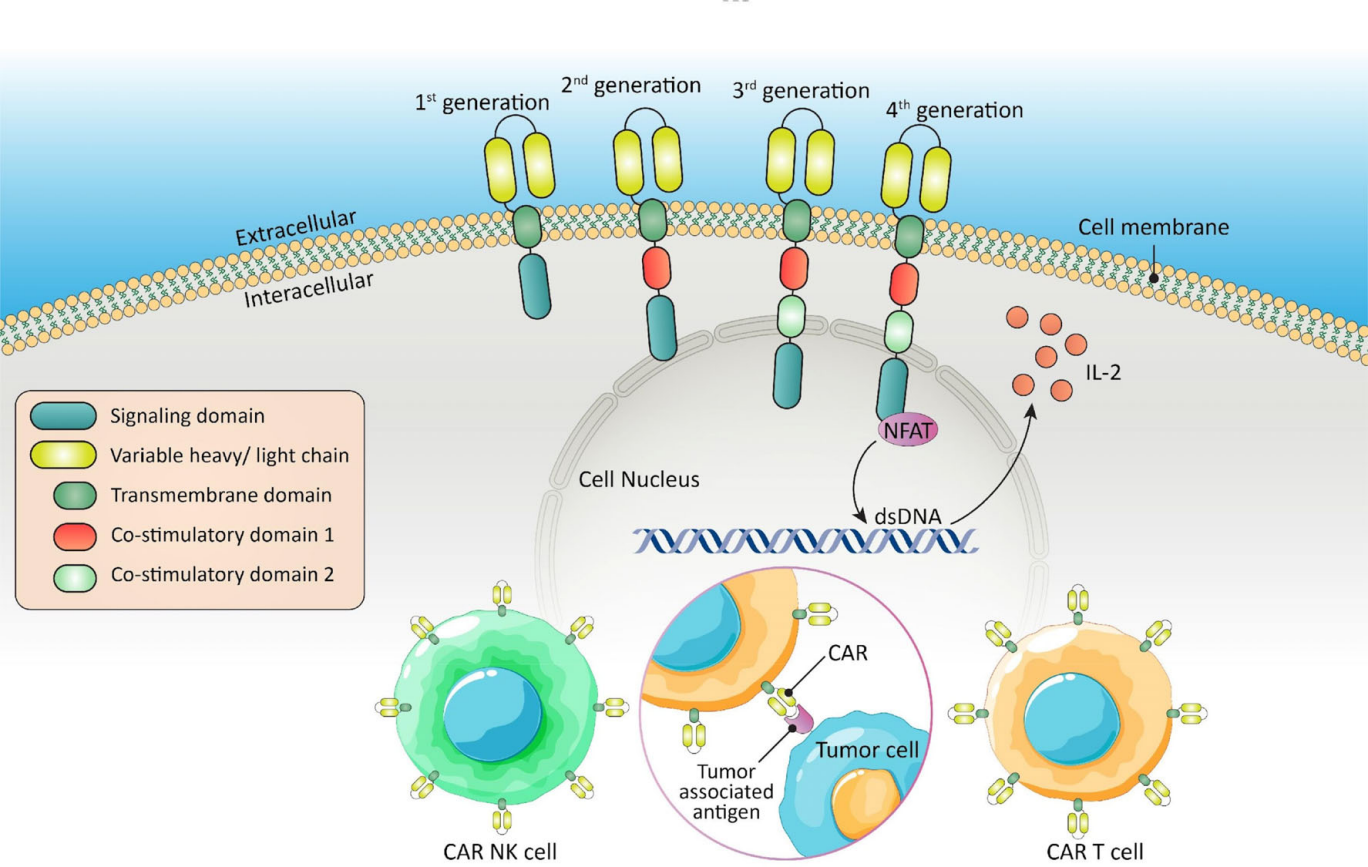
Different CAR T‐cell structural generations. Basic CARs can be divided into three regions: (i) the extracellular domain, which typically contains a single‐stranded variable fragment derived from an antibody; (ii) the transmembrane domain, which serves as an anchor support for the plasma membrane; and (iii) the signaling domain, which is responsible for cell activation. Only a signaling module derived from CD3 is present in the signaling domain of first‐generation CARs. A co‐stimulatory domain is also present in second‐generation CARs, and two co‐stimulatory domains are present in third‐generation CARs. TRUCKs, also known as fourth‐generation CAR T cells, are chemokine‐inducible cells that produce IL‐12.

Comprising three distinct domains—extracellular, transmembrane, and intracellular—these artificial receptors include an extracellular section with three components: a signal peptide, a region for recognizing TAAs, and a spacer.^90^ Within the extracellular domain, the section responsible for antigen binding is referred to as the scFv region. This region operates in a manner akin to the variable regions found in antibody VH and VL chains, connected by a flexible linker.^90^, ^91^ The spacer serves as a bridge between the antigen recognition region and the transmembrane domain, which takes the form of an alpha helix situated within the cell membrane. This transmembrane segment acts as a link connecting the extracellular antigen‐binding domain to the intracellular cytoplasmic domain. Comprising the fourth part of the CAR, the intracellular signaling domain predominantly includes an activation domain and one or more co‐stimulatory domains.^91^ The vast majority of CARs use tyrosine‐based activation patterns derived from immunoreceptors on CD3 to activate CAR T cells. Nevertheless, signaling mediated by these motifs on their own is insufficient to create efficient T‐cell responses, which has an insignificant effect on T‐cell activity and persistence in vivo; a co‐stimulatory signal is required for optimal T‐cell function, in vivo persistence, and metabolism. With the intracellular incorporation of one or two co‐stimulatory domains from co‐stimulatory receptor families (such as CD28, 4‐1BB, ICOS, or OX40), functional augmentation was accomplished, resulting in second‐ and third‐generation CARs (Figure 3), respectively, with improved antitumor efficacy and expansion in vivo.^92^, ^93^

A fourth‐generation T cell‐based immunotherapy, known as TRUCKs, has been developed, incorporating recent advances in genetic alterations involving cytokine release and other costimulatory ligands. This approach enhances CAR‐T‐cell activity and proliferation by making them more resistant to the immunosuppressive TME. Additionally, immunostimulatory cytokines such as IL‐2 are used to stimulate and recruit the innate immune system to the tumor site.^94^, ^95^

CAR‐T cells have limitations, such as not being appropriate for all patients due to significant T‐cell lymphopenia, as well as their toxicity profile. In addition, the effectiveness of the CAR‐T cell's killing mechanism relies on the interaction of target antigens with the CARs. In the context of CAR‐T‐cell therapy, a diminished cytotoxic response can result from the loss of the target antigen. In general and according to the limitations in the use or cytotoxic ability of these cells, various research have been conducted or are being done to evaluate different methods with the aim of solving these problems. The development of “off‐the‐shelf” allogeneic CAR‐Ts with low alloreactivity was made possible by creating a faster and less personalized manufacturing process for autologous CAR‐expressing cells. However, due to the potential for GVHD, employing allogeneic CAR‐T cells would necessitate further genetic modifications to mitigate this risk, such as the removal of the TCR gene. Given the patient's unique clinical and disease‐related characteristics and the aforementioned CAR‐T‐cell therapy's limitations, a different strategy has been developed using engineered NK cells.^86^, ^96^, ^97^, ^98^

NK cells are innate lymphoid cells that are CD3− CD56+ and are essential for the host's defenses against pathogens and cancers. Unlike T cells, NK cells have the ability to kill altered cells without the need for previous antigen stimulation, and they are not restricted by the expression of MHC molecules on the target cell.^99^

CAR‐engineered NK cells for tumor immunotherapy have become the center of rapidly growing interest. The advantages of CAR‐NK cells over CAR‐T cells include improved safety (e.g., lack of or reduced CRS and GVHD), utilizing a variety of methods to stimulate cytotoxic function, and high feasibility for “off‐the‐shelf” CAR cell manufacturing.^100^, ^101^

To address the therapeutic effectiveness of CAR T/NK cells in treating glioblastoma, some preclinical studies have been carried out, and many clinical trials are now being conducted.

### 3.1 Clinical trials of CAR T/NK cells for glioblastoma

Since August 2017, when the FDA certified the first CAR‐T‐cell therapy for treating B‐cell ALL, there has been a rising interest in utilizing CAR T cell‐based therapeutic strategies for addressing solid tumors, such as glioblastoma.^102^ Clinical trials employing a range of CAR‐T designs are currently being conducted to examine the safety and effectiveness of CAR‐T cells in glioblastoma treatment. Accordingly, the search results in ClinicalTrials.gov (https://clinicaltrials.gov/ct2/home) indicated more than 30 clinical trials for glioblastoma therapy using CAR T/NK cells registered until February 2023. In these trials, various tumor antigen targets, including HER2, EGFRvIII, IL13Ralpha2, CLTX, B7‐H3, IL‐8 receptor, NKG2D, IL7Ra, and CD147 were evaluated for the treatment of glioblastoma with first, second, third, and fourth generation CAR cells employing different integrated therapy approaches. Moreover, owing to the importance of a high transmission rate and stable gene expression in the production of CAR cells, retroviral and lentiviral vectors have been used extensively in these clinical trials. HER2, EGFRvIII, and IL13‐R2 can be regarded as safe and efficient targets for CAR therapy in the treatment of glioblastoma, according to the results of trials completed and published so far.^103^, ^104^, ^105^, ^106^

HER2, a transmembrane receptor tyrosine kinase, is found in approximately 80% of GBM cases. Consequently, HER2 becomes a promising target for CAR‐based immunotherapy in patients with HER2‐expressing GBM.^107^ Ahmed et al., in a phase I clinical study (NCT01109095) using bispecific anti‐HER2‐CMV CAR T cells in CMV‐seropositive patients, indicated that administering up to 1 × 10^8^ CAR T cells did not cause dose‐limiting toxicities and that eight of the 17 patients who received the treatment experienced an objective response.^104^

Additionally, preclinical studies supported the effectiveness and security of CAR‐NK cells based on second‐ as well as third‐generation CARs.^108^ The CAR2BRAIN interventional clinical trial (NCT03383978, clinicaltrials.gov) is the first and only CAR‐NK cell trial currently underway in Germany. The research revolves around the examination of a clonal product, ErbB2‐CAR‐NK‐92, intended for patients with glioblastoma.^109^ This phase I study, conducted across multiple centers and with an open‐label design, seeks to appraise the safety and acceptability of introducing genetically modified NK‐92/5.28.z cells into the brain using a lentiviral vector. The participants have recurrent HER2‐positive glioblastoma. The primary goal of this trial is to assess the safety and tolerance of NK‐92/5.28 thoroughly.z CAR‐NK cells designed to target ErbB2 through intracranial administration.^109^ A study in its initial stage treated six patients with recurrent glioblastoma using CAR T cells that targeted EGFR and IL13Rα2 through intrathecal delivery (_NCT05168423_). Treatment with CAR T cells resulted in neurotoxicity which was controlled with dexamethasone and anakinra (anti‐IL1R). Tumor size decreased, but there were no visible criteria for ORR. Initial findings suggest safety and bioactivity, with possible effectiveness that requires additional validation involving a larger number of patients and longer monitoring periods.^110^ The trial's findings are expected to be publicly available by 2023 (Table 1).

**TABLE 1:** Clinical trials of CAR T/NK cells for glioblastoma.

	Target antigen	Phase	Status	Treatment	Study start	Trial number
CAR‐T	HER2	I	Completed	CMV‐specific, HER2‐CAR T Cell	2010	NCT01109095
	IL‐13Ra2	I	Completed	IL13Ralpha2‐CAR T	2002	NCT00730613
	EGFRvIII	I/II	Completed	EGFRvIII‐CAR T‐cell Aldesleukin+Fludarabine+ Cyclophosphamide	2012	NCT01454596
	EGFRvIII	I	Completed	EGFRvIII‐CAR T‐cell +Pembrolizumab	2019	NCT03726515
	IL7Ra	I	Not yet recruiting	Truncated IL7Ra‐modified CAR T‐cell(Tris‐CAR‐T)	2023	NCT05577091
	CLTX	I	Not yet recruiting	CLTX‐CAR T‐cell(CHM‐1101 CAR‐T)? (CLTX(EQ)28ζ/CD19t + CAR T cells)	2023	NCT05627323
	B7‐H3	I	Recruiting	B7‐H3‐CAR T‐cell	2022	NCT05241392
	EGFRvIII	I	Recruiting	EGFRvIII‐CAR T‐cell + Cyclophosphamide+ Fludarabine	2016	NCT02844062
	IL‐8 receptor	I	Not yet recruiting	IL‐8 receptor‐modified CD70 CAR T‐cell(8R‐70CAR)	2022	NCT05353530
	NKG2D	NA	Not yet recruiting	NKG2D CAR‐T	2021	NCT04717999
	IL13Ralpha2	I	Recruiting	IL13Ralpha2‐CAR T + nivolumab + ipilimumab	2019	NCT04003649
	EGFRvIII	I	Active, not recruiting	EGFRvIII‐CAR T‐cell (EGFRvIII‐specific hinge‐optimized CD3 ζ‐stimulatory/41BB‐co‐stimulatory CAR T)	2020	NCT05063682
	B7‐H3	I	Recruiting	B7‐H3‐CAR T Cell	2022	NCT05474378
	EGFRvIII	I	Terminated(Halting enrollment of this study)	EGFRvIII‐CAR T Cell	2017	NCT03283631
	IL13Ralpha2	I	Recruiting	IL13Ralpha2‐CAR T cell	2020	NCT04661384
	CLTX	I	Recruiting	Chlorotoxin‐CD28‐CD3z‐CD19t‐CAR T cell	2020	NCT04214392
	EGFRvIII	I	Terminated (Study funding ended)	EGFRvIII‐CAR T‐cell (CD28, 4‐1BB, and CD3ζ)	2017	NCT02664363
	NA	I	Unknown	Anti‐PD‐L1 CSR T‐cell + cyclophosphamide+ fludarabine	2016	NCT02937844
	B7‐H3	I	Recruiting	B7‐H3‐CAR T cell	2022	NCT05366179
	NKG2D	I	Recruiting	NKG2D‐based CAR T (CD8 hinge region and transmembrane region, 4‐1BB costimulatory region and CD3 zeta region)	2021	NCT05131763
	CD147	(Early)1	Unknown	CD147‐CAR T‐cell	2019	NCT04045847
	B7‐H3	I/II	Recruiting	B7‐H3‐CAR T‐cell + temozolomide	2023	NCT04077866
	B7‐H3	I	Recruiting	B7‐H3‐CAR T‐cell + temozolomide	2022	NCT04385173
	EGFRvIII	I	Terminated (Sponsor decision to terminate prior to completion to pursue combination therapies)	EGFRvIII‐CAR T cell	2014	NCT02209376
	EGFRvIII	NA	Not yet recruiting	CARv3‐TEAM‐E T cell	2021	NCT05024175
	IL13Ralpha2	I	Active, not recruiting	IL13Rα2‐specific, Hinge‐optimized, 41BB‐costimulatory chimeric receptor and a truncated CD19	2015	NCT02208362
	EGFRvIII	I	Not yet recruiting	CARv3‐TEAM‐E T cell	2023	NCT05660369
	EGFRvIII	I	Enrolling by invitation	Antigen‐specific IgT cells expressing immune modulatory genes	2017	NCT03170141
	Her‐2	I	Recruiting	Memory enriched T cells (HER2 (EQ) BBζ/CD19t+ T cells)	2018	NCT03389230
	EGFR epitope 806 and IL13Ra2	I	Not yet recruiting	CART‐EGFR‐IL13Ra2 cell+ Cyclophosphamide + Fludarabine	2023	NCT05168423
CAR‐NK	Her‐2	I	Recruiting	CAR2BRAIN (CD28− CD3ζ) + Ezabenlimab	2017	NCT03383978

## 4 ENHANCE CAR T/NK CELL EFFICIENCY BY TARGETING THE IMMUNOSUPPRESSIVE TME FOR GLIOBLASTOMA

Despite the significant results of targeting hematological malignancies, a number of major hurdles, such as heterogeneous, varied, and complex tumor antigen expression and immunosuppressive TME, have a negative impact on the accessibility, infiltration, stimulation, activation, and persistence of CAR‐expressed cells at the tumor site. These roadblocks have prevented this type of therapy from being successful in the fight against solid tumors.^98^ To establish an immunosuppressive environment and evade immunotherapy strategies, tumor cells recruit immunosuppressive cells. In addition, the TME's metabolic characteristics, co‐existence, and interaction of tumor cells with immunosuppressive cells promote tumor growth, suppress the immune system, and inhibit the anticancer effectiveness of infused CAR cells. The TME represents one of the major obstacles to the cytotoxic power of CAR cells because it prevents CAR cells from reaching the targeted site of action, interferes with their metabolic function, and produces an immunosuppressive environment that causes T‐cell exhaustion.^111^, ^112^

The TME of solid tumors functions as a physical impediment that obstructs the infiltration of CAR cells and effector T cells engaged in tumor infiltration, employing diverse mechanisms. A key obstruction arises from forming a robust fibrogenic TME orchestrated by stromal cells such as CAFs. Upon activation by TGF‐b, these cells facilitate the generation of ECM proteins that curtail the mobility and migration of T cells.^113^, ^114^ The effectiveness of cancer immunotherapy and the accomplishment of T cells infused for their anti‐tumor properties hinge on the successful infiltration of T cells into the tumor stroma. Compared to hematological malignancies, solid tumors are less accessible and more difficult to treat, and extracellular matrix and tumor vasculature act as major physical obstacles that prevent infused‐CAR T‐cell penetration to tumor tissue. Therefore, identifying the barriers to T‐cell trafficking and designing strategies to overcome these obstacles could increase the efficiency of CAR cells in vivo. ECM component expression and density have both risen, particularly hyaluronan and collagen, in tumor tissue and limit the penetration of therapeutic agents.^115^, ^116^ Examination of T‐cell movement and positioning within the tumor stroma unveiled an inverse relationship between T‐cell infiltration and the stiffness of the ECM. T cells have a tendency to gather in areas characterized by reduced fibronectin and collagen density.^117^

**FIGURE 4:**
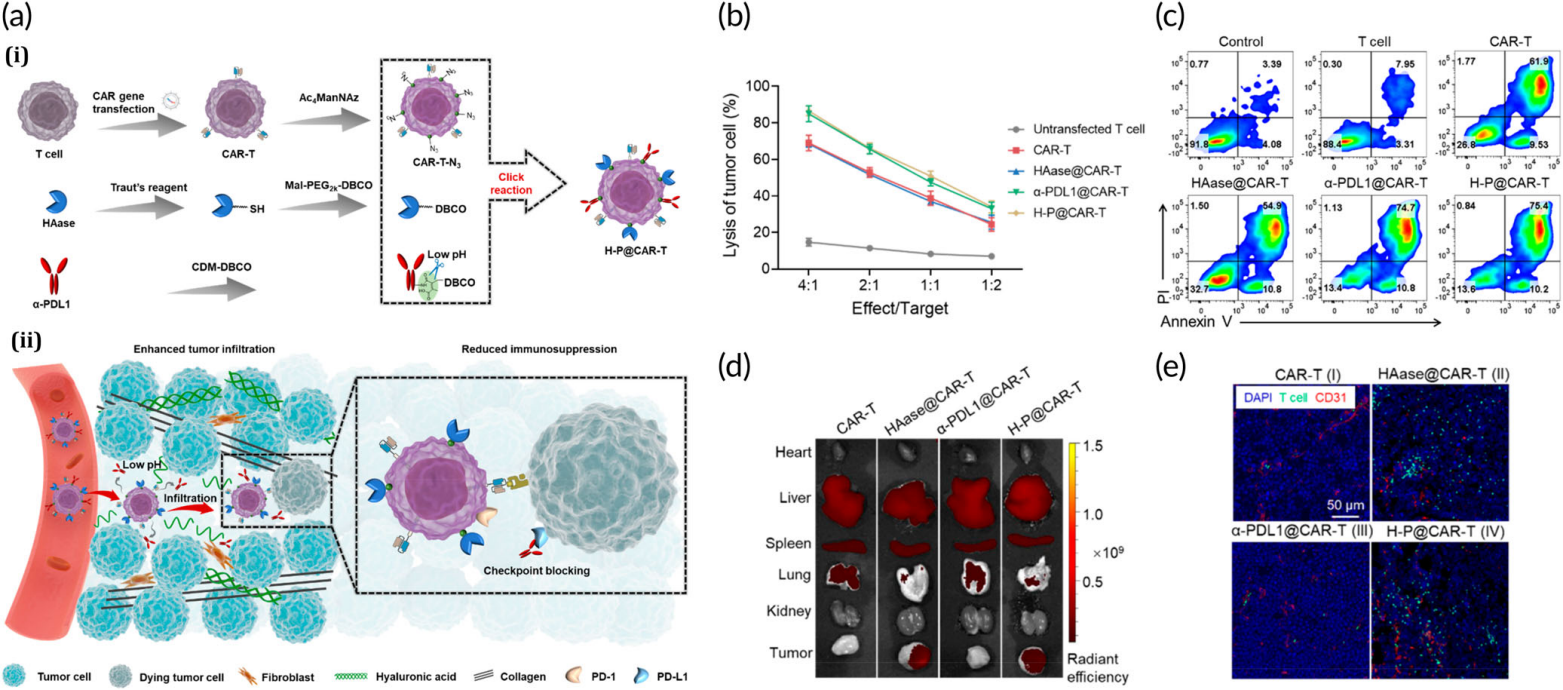
A chemical approach to modify conventional CAR‐T cells with the intention of increasing their effectiveness as a treatment for solid tumors. (a) Schematic of H‐P@CAR‐T cells for improved solid tumor immunotherapy, which were engineered with HAase and α‐PDL1. (i) Tumor ECM degrading enzyme HAase and checkpoint blocking antibody α‐PDL1 have been developed on the CAR‐T‐cell surface by metabolic glycan biosynthesis and click reaction. Maleic acid amide bonds were conjugated to α‐PDL1 so that it could be released in a low pH TME and respond to extracellular acidity in tumors. (ii) Hyaluronic acid degradation by H‐P@CAR‐T cells results in the destruction of the tumor's extracellular matrix and accelerated tumor invasion. In addition, the tumor microenvironment's acidic conditions cause the checkpoint‐blocking antibody α‐PDL1 to release from H‐P@CAR‐T cells, reversing the immunosuppression of the PD1‐PDL1 pathway and enhancing antitumor activity. (b) Tumor cytotoxicity of engineered CAR‐T cells on A20 tumor cells (*n* = 3). (c) Analysis of apoptosis in A20 tumor cells following a 24‐h incubation with the T lymphocytes and CAR‐T cells. (d) Ex vivo fluorescence images of the modified CAR‐T‐cell biodistribution and tumor accumulation after 24 h following delivery of various CAR‐T cells. (e) evaluating the intratumor distribution of CAR‐T cells using stained sections of tumor tissues that were obtained 24 h after the injection. CAR‐T cells (green) that were CFSE‐labeled were injected intravenously into A20‐bearing mice. The blood vessels within the tumor were stained with α‐CD31‐PE (red). Reproduced from Zhao et al.^121^ under open access license.

Moreover, higher collagen density hampers T‐cell proliferation and cytotoxic activity while promoting a regulatory phenotype.^116^ To enhance the delivery of antitumor therapeutic agents, using ECM‐degrading enzymes like hyaluronidase and collagenase has been proposed to reduce ECM rigidity.^117^, ^118^ Based on these discoveries, Lokeshwar and his team took the initiative to induce the synthesis of HPSE in T cells modified with CARs. Co‐expressed CAR T cells were more capable of degrading the ECM as a result of this augmentation, thereby enhancing their effectiveness in combating solid tumors.^119^ Furthermore, the expression of HAS has shown an adverse association with patient survival across diverse solid tumors, including gastric cancer. A recent study examined how co‐expressing a secreted form of human hyaluronidase PH20 affected the ability of anti‐mesothelin CAR‐T cells to degrade HA. In vitro, HA hindered the mobility of CAR‐T cells, thereby inhibiting their anti‐tumor activity against gastric cancer. Researchers then developed a secreted form of human hyaluronidase PH20 using IgG2 Fc fragments instead of the PH20 signal peptide. The study demonstrated that overexpressing sPH20‐IgG2 increased CAR‐T‐cell transmigration through an HA‐containing matrix but did not affect their cytotoxicity or cytokine release. Moreover, administering sPH20‐IgG2 resulted in enhanced infiltration of anti‐mesothelin CAR‐T cells into gastric cancer cell xenograft tumors.^120^ To boost the therapeutic impact on solid tumors, scientists modified CAR‐T cells by incorporating HAase and the checkpoint‐blocking antibody α‐PDL1 on their surface. The in vitro infiltration assays and in vivo biodistribution studies revealed that the engineered HAase effectively breaks down hyaluronic acid and removes the tumor's extracellular matrix, enabling CAR‐T cells to infiltrate the solid tumors more efficiently. Moreover, in vitro cytotoxicity experiments demonstrated that CAR‐T cells expressing α‐PDL1 exhibited more potent antitumor activity than conventional CAR‐T cells. Importantly, HAase‐ and α‐PDL1‐engineered CAR‐T cells improved treatment effectiveness on solid tumor models and exhibited minimal systemic side effects (Figure 4).^121^

Targeting cancer‐associated stromal cells using CAR T cells, bispecific antibodies, or chemokine inhibition could offer significant advantages for CAR T/NK cell therapies. Notably, CAFs play a crucial role in constructing and remodeling the TME. Through the secretion of ECM proteins and various regulatory factors influencing tumorigenesis, angiogenesis, and metastasis, CAFs promote tumor initiation, growth, and metastasis.^122^, ^123^ Enhancing immunotherapy strategies can involve depleting or modifying CAF activity, leading to reduced CAF numbers, which may synergistically enhance CAR T/NK cell therapy by promoting lymphocyte migration into the tumor mass. Furthermore, several CARs have been developed to specifically target and deplete CAFs through the FAP‐α. While research by Schuberth et al. showed limited anti‐tumoral effects of anti‐FAP‐α CAR T cells and significant “on‐target, off‐tumor” toxicity, anti‐FAP‐α CAR‐T‐cell therapy has shown promising effectiveness in numerous preclinical models of various solid tumors.^124^, ^125^

Furthermore, ligands for NK cells' endogenous activating receptors might be downregulated by solid tumors, which further reduces their anti‐tumor effectiveness.^126^ Genetic modification of NK cells to express CARs provides strong activating, co‐stimulatory, and cytokine signals. The goal is to counteract inhibitory signals from the TME and shift signals in favor of NK cell activation. However, despite positive outcomes in preclinical studies against different solid tumor models, most of these studies lacked TME components. As a result, there is currently no evidence of CAR NK cells being effective in challenging TMEs.^127^, ^128^, ^129^, ^130^

### 4.1 Tumor vasculature

New blood vessels must develop in order for cancer cells, which undergo uncontrolled cell division, to receive nutrition and oxygen. Abnormal vasculature development and reduced expression of adhesion molecules, driven by angiogenic factors like bFGF and VEGF, restrict the entry of T cells into the tumor region.^131^ CAR T cells must effectively extravasate through solid tumor vasculature and penetrate the tumor tissue in order to target specific antigens on the tumor cells. The success of T‐cell therapies largely hinges on the intricate process of T‐cell trafficking, which includes various stages like rolling, adhesion, extravasation, and chemotaxis. T cells are equipped with active ligands that can interact with selectins, facilitating their rolling motion along the surface of ECs.^132^, ^133^ In the rolling phase, chemokine receptors present on the surface of T cells engage with chemokines located on ECs. This interaction initiates internal signaling pathways that activate adhesion molecules from the integrin family both on the T cells and the ECs. Subsequently, additional chemokine interactions lead to T‐cell diapedesis and extravasation. These integrins bind to ICAM‐1 and VCAM‐1 on the endothelial cells, promoting a strong attachment. The term “endothelial cell anergy” pertains to the general inhibition of these adhesion molecules on endothelial cells within the tumor's vasculature. The tumor blood vessels' tortuous structure, characterized by dilatation and leakiness, causes heterogeneous permeability, raised interstitial pressure, and irregular blood flow, all of which further impact immune cell infiltration and function.^134^ Moreover, within the TME, endothelial cells induce the production of inhibitory molecules such as FasL, PD‐L1, TIM3, IDO‐1, PGE2, and IL‐10, which hinder the activity of effector T cells. The unique features of tumor blood vessels are influenced by the presence of VEGF and the overexpression of VEGFRs.^135^ CARs designed to target VEGFR1 and VEGFR2 have demonstrated potential in eradicating tumor vasculature, limiting tumor cell proliferation, and impeding the supply of nutrition and oxygen to tumors.^135^, ^136^ Recent research has shown that tumor‐specific immunotherapy can be significantly improved by co‐infusing VEGFR2‐specific CAR T cells and antigen‐specific TCR‐transduced T cells together. This combined approach enhances the infiltration, persistence, and anticancer activity of tumor‐specific T cells, leading to increased effectiveness in combating cancer.^137^ TNBC has been reported to be treated with CAR immunotherapy that targets TEM‐8. After the CAR‐T‐cell infusion, the tumor size decreased significantly. After 2 months of treatment, tumors remained noticeably smaller and contained noticeably fewer blood vessels, even though the TNBC xenografts were not entirely eradicated.^138^ Xie et al. described an innovative strategy by targeting the fibronectin splice variant that contains the EIIIB domain, which is observed in the angiogenic vasculature of tumors. This strategy was not successful in mice with immunodeficiency, indicating the important role of endogenous immunity in CAR‐based therapies.^139^ Fu et al. studied the use of CAR T cells to target v3 integrins expressed on tumor endothelial cells to reduce tumor vasculature using an RGD domain‐based mediated targeting method. The CAR utilized a modified ectodomain of the polypeptide echistatin sourced from the venom of the viper (*Echis carinatus*). This component exhibited a strong affinity for the v3 integrin found on tumor‐associated endothelial cells. When applied in a mouse model of melanoma, these v3 integrin‐targeted CAR T cells damaged the tumor‐associated blood vessels, resulting in the elimination of the tumor mass. However, it is worth noting that this approach also carried the risk of potential side effects, such as tumor‐site hemorrhage.^140^

Approximately 50% of glioblastoma patients have tumors that express wtEGFR, and in a smaller percentage of instances, both wtEGFR and the mutant form EGFRvIII. However, studies using CAR T cells that have been previously described only target EGFRvIII. Recently, CAR‐redirected NK cells can successfully target wtEGFR and EGFRvIII to treat glioblastoma. Lentiviral constructs carrying a second‐generation CAR designed to target both wtEGFR and EGFRvIII were employed to transduce human NK cell lines, namely NK‐92 and NKL, as well as primary NK cells. When these EGFR‐CAR‐engineered NK cells were co‐cultured with GB cells or patient‐derived GB stem cells, they exhibited enhanced cytolytic activity and increased production of IFN.^129^

### 4.2 Chemokine expression profile as an effective system for directing CAR T/NK cells into solid tumors

**FIGURE 5:**
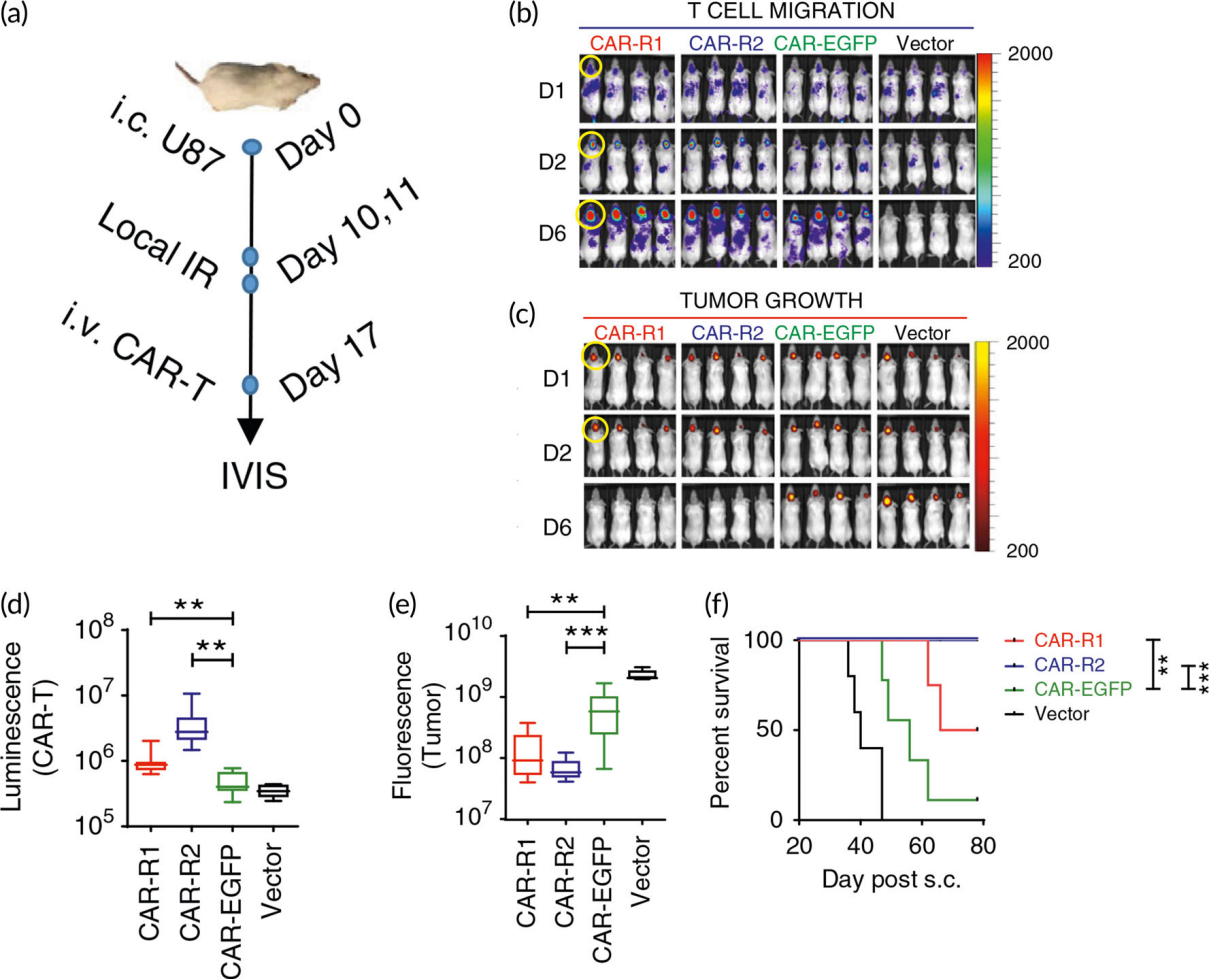
In pre‐clinical GBM models, IL‐8 receptor, CXCR1 or CXCR2, modified CARs significantly increase T‐cell migration and persistence in the tumor, leading to full tumor regression and long‐lasting immunologic memory. (a) On Day 0, the treatment plan was started with intravenously 5 × 10^4^/mouse i720 transduced U87 tumor cells injection. On Days 10 and 11, fractionated local IR was administered (at a rate of 4.5 Gy/day), and on day 17, CARs or vector T cells were administered. T‐cell luminescence and tumor fluorescence images were captured using the IVIS system (Xenogen, Alameda, CA). (b, c) Tumor development and T‐cell migration at an ROI after 1, 2, and 6 days following CAR T‐cell therapy (yellow circles indicate ROI). (d) T‐cell migration to the ROI after the T‐cell transfer, (e) quantification of tumor growth 10 days after T‐cell transfer, and (f) survival rate (*n* = 4–5/group). Reproduced from^143^ under open access license.

It has been demonstrated that harnessing the chemokine system, which plays a significant role in hematopoietic cell migration, is an attractive strategy to increase the therapeutic activity of CAR‐T cells by increasing their trafficking into tumors. The chemokine receptor axes have been investigated as a method to improve intra‐tumoral T‐cell trafficking. It may be possible to determine which chemokine axis can be used to improve CAR‐T‐cell trafficking into solid tumors by understanding the expression profile of chemokines in the TME. The directed migration of CAR T cells is known to be influenced by engineered T cells by specific chemokine receptors according to chemokines produced by tumors. Integrin v6‐CAR T cells that have been engineered to express CXCR2 migrate more efficiently towards IL‐8 generated by tumors.^141^ In different solid tumor xenografts, CAR T cells overexpressing CXCR1 or CXCR2 can considerably reduce tumor burden without noticeable toxicity (Figure 5).^142^, ^143^

Furthermore, the expression of CXCL9, CXCL10, and CXCL11 (ligands of CXCR3), CCR4, and CCR2b enhances T‐cell trafficking to the TME.^144^, ^145^, ^146^

Furthermore, the limited capacity of CAR NK cells to infiltrate the tumor site hinders the successful eradication of solid tumors. Achieving effective infiltration of NK cells into the tumor sites is vital for the success of CAR NK cell therapy. To address this issue, numerous scientists have modified CAR NK cells by incorporating chemokine receptors. This modification aims to amplify the migration of NK cells toward tumor locations where the relevant chemokines are expressed. Consequently, this strategy leads to an augmented trafficking of NK cells to the tumor regions. Notably, glioblastoma exhibits elevated levels of the chemokine CXCL12/SDF‐1, primarily expressed in bone marrow stromal cells. To improve the targeting of CAR‐NK cells in the bone marrow and their entry into glioblastoma tumor sites, researchers have looked to integrate the CXCR4 receptor into CAR constructs. When compared to using only anti‐EGFRvIII CAR‐NK cells, the approach of employing CXCR4‐engineered anti‐EGFRvIII CAR‐NK cells has shown enhanced migration towards glioblastoma sites that produce the specific chemokine CXCL12/SDF‐1. This approach has led to complete cancer remission and enhanced survival outcomes.^147^ To avoid negative side effects, it is important to consider the impact of abnormal chemokine receptor signaling in T cells. New techniques for tumor induction to express the required chemokine ligands are being investigated. Alternatively, local chemokine production or delivery could be used to enhance CAR T‐cell migration and tumor infiltration.^148^, ^149^, ^150^, ^151^, ^152^

## 5 COMBINATION THERAPIES FOR BOOSTING CAR CELLS IN GBM IMMUNOSUPPRESSIVE TUMOR MICROENVIRONMENT

Recent studies have highlighted the potential of employing oncolytic viruses equipped with chemokines to enhance the immune functionality of CAR T cells within solid tumors. Both oncolytic viruses and CAR T cells possess inherent cytolytic capabilities, making their combined usage a promising strategy for optimizing the advantages of each approach while mitigating their limitations.^153^ Notably, Wang et al. observed that the combination of CAR‐T cells with an oncolytic virus carrying RANTES chemokine and IL‐15 cytokine led to an immediate lytic effect on infected malignant cells. Additionally, this approach resulted in heightened migration and prolonged survival of CAR‐T cells.^154^

Due to challenges related to limited migration following intravenous administration, researchers have explored the option of administering CARs through local infusion. In ongoing clinical trials (NCT02498912, NCT02414269, NCT01818323), a comparison is being made between the advantages and disadvantages of systemic versus localized infusion of CAR T cells in diverse solid tumors. One potential drawback is that, particularly for brain tumors, local administration is typically more technically difficult than direct intravenous administration. There is evidence that CAR‐T therapy may be more efficient when used in combination with other treatments such as radiation, chemotherapy, bevacizumab, checkpoint inhibitors, oncolytic virotherapy, TKIs, and CRISPR/ CAS9 technologies.^154^, ^155^ According to a recent study, the GBM TME undergoes considerable modifications prior to chemotherapy and radiotherapy. These changes result in an improved anti‐tumor response, upregulation of tumor antigens, and the elimination of immunosuppressive cells.^156^

In animal models of GBM, Suryadevara et al. discovered that CAR T therapy given after temozolomide treatment enhanced CAR T engraftment, proliferation, and expansion, leading to increased cytotoxic activity.^157^ In a recent clinical study, the anti‐tumor efficiency of combination immunotherapy with IL13Ralpha2‐CAR T cell, nivolumab, and ipilimumab on GBM is under investigation (NCT04003649). Furthermore, researchers assessed the cytotoxic potency of EGFRvIII‐directed CAR T cells when combined with pembrolizumab (a PD‐1 inhibitor) for MGMT‐Unmethylated Glioblastoma (NCT03726515).

Radiotherapy has the potential to alter the GBM TME, leading to increased efficacy of CAR T cells by creating favorable niches for their proliferation. Firstly, radiation directly targets and kills tumor cells, resulting in changes in the expression of tumor‐specific antigens due to BBB damage and increased permeability.^158^, ^159^ This leads to an augmented expression of CAR targets on the tumor and enhanced activation of APCs, enabling cytotoxic CD8+ T lymphocytes to recognize and eliminate malignant cells more effectively.^160^, ^161^ Secondly, exposure to ionizing radiation can trigger the release of chemokines such as CXCL9, CXCL10, and CXCL16, along with proinflammatory cytokines like IFN‐γ, which promote the recruitment of T cells.^161^

The combination of CAR T‐cell treatment with radiation has demonstrated increased effectiveness in GBM models and various other solid tumors. Similarly, improved immune cell infiltration and CAR‐T‐cell activity have been observed in murine GBM models when they combined radiation with NKG2D CAR T cells.^162^

TKIs are a promising option for CAR T‐cell treatment in solid malignancies. LB‐100, a small molecule inhibitor that targets PP2A involved in cell–cell adhesion, can enhance the efficacy of CAIX‐specific CAR‐T‐cell therapy in both in vivo and in vitro models of GBM.^163^, ^164^

### 5.1 Targeting hypoxia in the tumor microenvironment

A majority of malignant tumors experience hypoxia, a non‐physiological level of oxygen concentration. Because of progressive yet inefficient vascularization and the adoption of the epithelial‐to‐mesenchymal transition phenotype, metastasis, and cell mobility are caused by tumor hypoxia. By inducing cell quiescence, hypoxia modifies the metabolism of cancer cells and contributes to the development of drug resistance. The HIF, PI3K, MAPK, and NF‐B pathways are only a few of the complex cell signaling networks that hypoxia activates in cancer cells. These pathways interact with one another to create positive and negative feedback loops, which can either increase or decrease the effects of hypoxia.^165^, ^166^, ^167^ The hypoxic microenvironment plays a vital role in angiogenesis, invasion, survival, and resistance to therapies in solid malignancies. Moreover, tumor hypoxia is directly associated with disease progression and poor prognosis in patients with glioma. It can lead to the production of unique proteins that can promote tumor growth and advancement.^168^ Exploiting the unique molecular aspect of tumor hypoxia is a promising strategy in cancer treatment called hypoxia‐targeting gene therapy. Upregulating HIFs is a critical response of tumor cells to hypoxia, which further regulates gene transcription by binding to HRE regions, leading to cellular adaptation to environmental changes and cell survival.^169^, ^170^ The PHD2 detects changes in oxygen tension (pO_2_) and regulates the expression of HIFs.^171^ The low oxygen tension during hypoxic conditions inhibits PHD2, which causes HIF‐1 accumulation. The VEGF, FGF, and the other proangiogenic genes are upregulated as a result of HIF‐1 and HIF‐1 dimerization, which causes a rise in tumor migration, invasiveness, and angiogenesis.^172^ Several studies have demonstrated the anti‐tumor effect of the downregulation of PHD2 in GBM as a potential therapeutic target, which plays an indispensable role in tumor progression and HIF regulation.^173^, ^174^ Two clinical trials, NCT04874506 and NCT02974738, demonstrate the inhibition of HIFs in GBM patients using MBM‐02 (Tempol) and Belzutifan.^173^ Conversely, TCR activation signals or cytokines produced during infection and inflammation can regulate HIF synthesis and stability, impacting T lymphocytes, CAR activation, and differentiation.^169^ Furthermore, the localization of CAR T cells in the hypoxic TME can influence their effectiveness, and understanding the responsible mediators can contribute to the development of more potent CAR‐engineered T cells. In a study from 2017, researchers developed an oxygen‐responsive CAR by combining the oxygen‐sensitive domain of HIF1α with the intracellular CAR domain's C‐terminal end. This approach aimed to restrict CAR presentation and activation to the hypoxic region, thereby reducing on‐target/off‐tumor toxicity.^175^ On the other hand, targeting antigen overexpression, like CAIX in glioblastoma, presents a promising approach to redirect CAR‐T cells for recognizing and eliminating cancer cells.^176^ However, in the TME, interactions between hypoxia‐derived accumulated adenosine and specific receptors on T‐cell surfaces (A2AR and A2BR) can disrupt TCR signaling and hinder T‐cell antitumor activity. Conversely, inhibiting PD1 interaction with PDL1 led to increased A2AR expression on CD8+ T lymphocytes infiltrating the tumor, making them more susceptible to immunosuppression by accumulated adenosine. Consequently, targeting both the PD1/PDL1 axis and adenosine A2A receptors simultaneously (through genetic or pharmacological methods) may enhance T‐cell performance in CAR‐T‐cell therapy.^176^, ^177^, ^178^

The HiTA system is designed by He group. This system can be used to regulate specific CARs for different tumor antigens or any immunomodulator in the tumor lesions. This HiTA system demonstrated significantly improved hypoxia‐restricted transgene expression compared to previous approaches and effective antitumor activity without in vivo evidence of significant liver or systemic toxicity. This method can be used to create CAR T cells that target different tumor antigens.^165^

### 5.2 The immune checkpoints

Lately, the inhibition of ICs, a group of pathways that regulate the immune system's function by providing inhibitory signals to T cells, has emerged as an advanced cancer therapy strategy. Although this mechanism prevents autoimmunity, it can hinder the immune response against cancer cells.^179^, ^180^ Various checkpoint molecules play a significant role in immunosuppression, including the essential ICs: PD‐1 and its ligand (PD‐L1), CTLA‐4, and mucin domain‐3 (Tim‐3).^180^ A key immune checkpoint molecule involved in GBM immunity escape is PD‐L1, expressed on GBM tumor cells, microglia, and TAM, binds to PD‐1 to downregulate the T‐cell mediated immune response, thereby suppressing the proliferation and function of cytotoxic T cells and promotes T‐reg activity.^181^, ^182^ In addition, exhausted CD8+ T cells overexpress immune checkpoint receptors, and blockage of signaling pathways of these receptors has been indicated to improve the early stages of T‐cell exhaustion.^183^ Despite the remarkable success of the ICB observed in various cancers, the same effect is not reported in GBM.^184^, ^185^ PD‐L1 expression in glioblastoma patients is heterogeneous and rare in tumors and peripheral immune cells.^185^, ^186^ Exclusively, glioma‐infiltrating MDMs express lower levels of PD‐L1 than MDMs that infiltrate brain metastases.^185^, ^187^ Although some pre‐clinical investigations achieve brilliant results,^188^, ^189^ this could explain the lack of association between PD‐L1 expression and survival in clinical trials evaluating ICB for GBM.^190^, ^191^, ^192^ The promising outcomes have positioned this immune checkpoint as a primary target for GBM immunotherapy. Another crucial immune checkpoint molecule, CTLA‐4, expressed on both activated T cells and Tregs, could also play a pivotal role in GBM immune evasion. This molecule interacts with CD80 and CD86 on APCs to inhibit co‐stimulatory pathways of T cells.^193^, ^194^ Blocking CTLA‐4 by CTLA‐4 inhibitors such as ipilimumab and tremelimumab may potentially enhance the anti‐tumor activity of T cells.^195^

Despite these treatment strategies, checkpoint blockade therapies have not been successful against GBM solitary. Recently, combination therapy has been highlighted as a more efficient strategy for GBM and other solid tumors. The use of CAR T cells and checkpoint blockage combination is one of the most cutting‐edge immunotherapy combinations. Through employing this approach, researchers expect to minimize the effects of tumor heterogeneity and T‐cell depletion.^196^ Recently, different preclinical animal studies have been done in murine and canine GBM models by focusing on combining CAR T cells with ICB approaches in these tumors. It is proven that CAR T cells specific for IL‐13Rα2 antigen can become exhausted in GBM‐bearing hosts, and researchers believe that blockage of inhibitory surface antigens can be the best strategy.^197^, ^198^, ^199^ Sengupta et al. demonstrated that T‐cell proliferation increased and exhaustion of T cells reduced by decreasing PD‐1 levels resulting in the treatment with GSK3‐inhibited CAR‐T cells. This protein, which is constitutively active in T cells, controls the proliferation of naive T cells.^197^ Another research group generated two CAR‐T cells targeting the human IL‐13Ra2 receptor. In a subcutaneous human glioblastoma xenograft model, these two engineered cells exhibited excellent tumor suppressive activity when compared to the humanized CAR‐T construct EGFRvIII used in a recent phase 1 clinical trial (NCT02209376). Furthermore, they demonstrated that combining these humanized IL‐13Ra2 CAR T‐cells with CTLA‐4 inhibition significantly enhanced their effectiveness. In the same mouse model of GBM, the efficacy of humanized CAR T‐cells was further improved by PD‐1 and TIM‐3 blockade.^196^, ^200^ Numerous clinical trials evaluating CAR‐T‐cell therapy with checkpoint blockade in Glioblastoma are currently listed (Table.2).

**TABLE 2:** Clinical trials evaluating CAR T‐cell therapy with checkpoint blockade in Glioblastoma.

NCT Number	Checkpoint inhibition strategy	CAR T cell	Malignancy	Year (reference)
NCT03726515	CAR T‐cell plus αPD‐1 mAb Pembrolizumab	EGFRvIII CAR	EGFRvIII+ Glioblastoma	2019^220^
NCT04003649	CAR T‐cell plus αPD‐1 mAb Nivolumab and αCTLA‐4 Ipilimumab	IL‐13Rα2 CAR	IL‐13Rα2+ Glioblastoma	2022^221^
NCT02873390	CAR T cell Expressing αPD‐1 mAb	EGFR CAR	EGFR+ cancers	2017^222^
NCT03182816	CAR T cell Expressing αPD‐1 mAb and αCTLA‐4	EGFR CAR	EGFR+ cancers	2021^223^

## 6 THE INCORPORATION OF NANOTECHNOLOGY INTO CAR T/NK CELL THERAPY

The use of cell therapy in solid tumors, particularly in CNS tumors, faces challenges such as difficulty in intratumoral delivery and immunosuppressive tumor microenvironments. Nanoparticles offer potential solutions by enabling precise cell activation ex vivo. In addition, they can be designed to specifically encapsulate cell stimulating agents for co‐localized stimulation and lead to enhanced cell functionality. Nanotechnology has altered the landscape of CAR T/NK cell therapy by presenting various strategies to increase the effectiveness of these cutting‐edge immunotherapy tools. Recent preclinical advancements emphasize the role of nanoparticles in enhancing cell therapy and discuss their potential in treating CNS tumors.^201^, ^202^

Through innovative nanoscale materials and structures, researchers designed targeted delivery systems that can improve the specificity and precision of CAR T/NK cell therapies.^203^, ^204^ These nanocarriers can be engineered to encase CAR T/NK cells and deliver them directly to cancerous sites, reducing off‐target and maximizing therapeutic results. These vehicles can be customized and functionalized with ligands that specifically bind to precise receptors on tumor cells, allowing for accurate transportation of CAR T/NK cells to tumor sites. In addition, by enclosing CAR T/NK cells within these nanocarriers, it can be possible to protect CAR cells from the host immune system, prevent degradation in the bloodstream, improve their retention at the tumor site, minimize systemic toxicity and improve the safety profile of these therapies.^205^, ^206^

Furthermore, nanotechnology facilitates the development of smart drug delivery systems that can release CAR T/NK cells in a controlled manner, optimizing their function within the tumor microenvironment. By incorporating stimuli‐responsive nanoparticles or nanocarriers, researchers can design platforms that respond to specific cues in the tumor environment, such as pH or enzyme levels. This level of precision and control is vital for maximizing the therapeutic potential of CAR T/NK cell therapies while minimizing systemic toxicity.^206^, ^207^, ^208^

In addition to improving the delivery and targeting of CAR T/NK cells, nanotechnology also offers opportunities to boost the functionality of these engineered cells. Nanoscale platforms can be used to modulate the signaling pathways within tumor sites or CAR cells, enhancing their cytotoxic power and overcoming mechanisms of immune evasion. By incorporating nanomaterials that can interact with the immune cells at a molecular level, scientists can adjust and fine‐tune the behavior of CAR cells to improve their persistence and success in combating cancer.^209^, ^210^, ^211^

**FIGURE 6:**
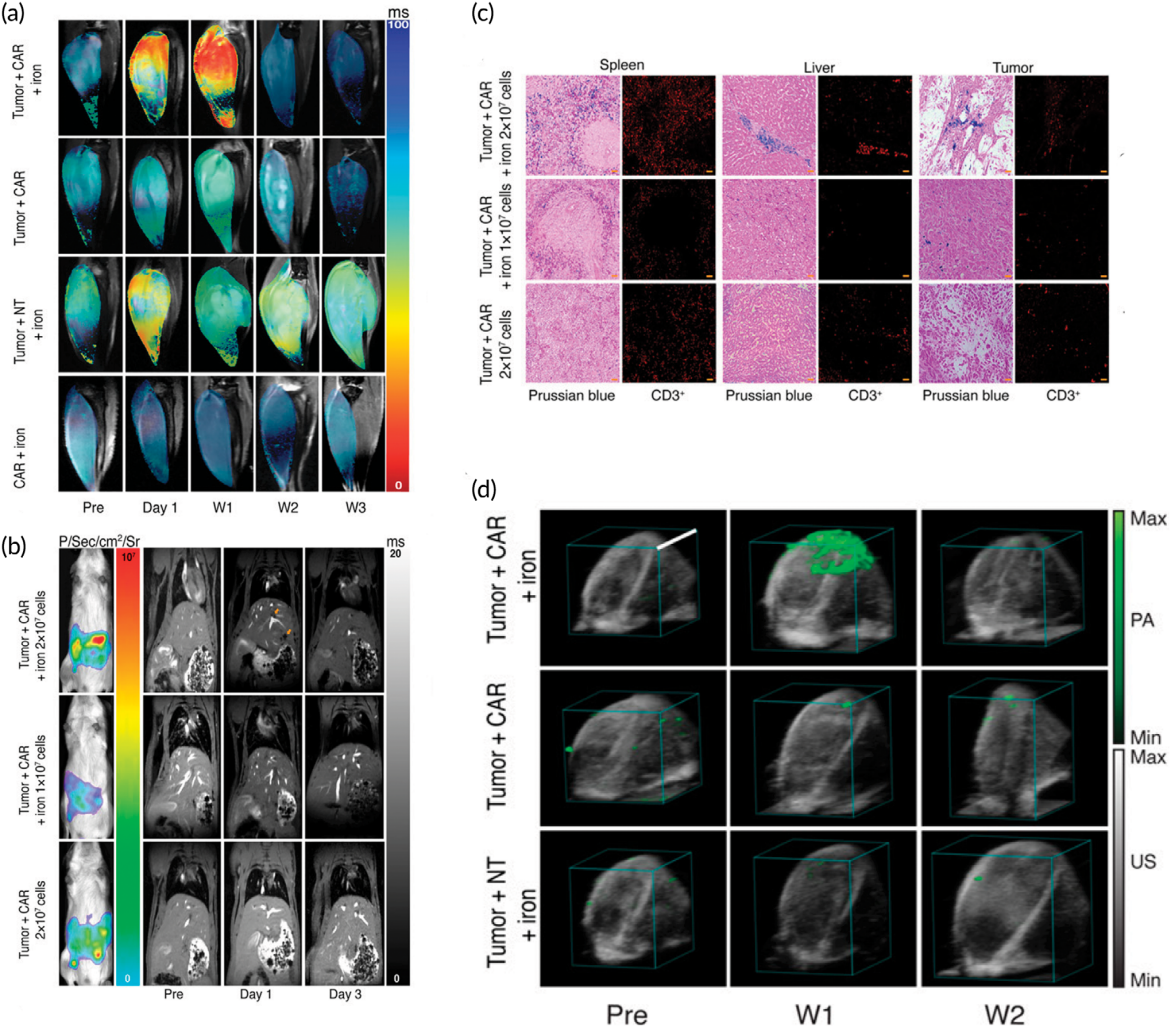
A clinically translatable method that can be used in clinical settings to label CAR T cells with iron oxide nanoparticles was developed. The labeled CAR T cells were then detected using MRI. (a) Representative coronal T2*‐weighted MR images of osteosarcomas in the tibia of experimental mice were shown, along with superimposed, color‐coded T2* maps. (b) Representative bioluminescence images acquired 1 h post‐injection demonstrated the viability of the anti‐B7‐H3 CAR‐nLuc T cells. Coronal gradient echo MR images of animals injected with B7‐H3 CAR‐nLuc T cells revealed the colocalization of the iron‐labeled CAR T cells at off‐target sites (orange arrows). (c) Representative histological images of paraffin‐embedded tissue sections obtained from mice on Day 3 post‐treatment stained with Prussian blue DAP (left) and the anti‐CD3 primary antibody and cy‐5‐conjugated secondary antibody (right) are shown. The scale bars represent 50 μm, with a magnification of ×20. (d) Visualization of CAR T cells labeled with ferumoxytol using photoacoustic tomography (PAT) and magnetic particle imaging (MPI) is presented. Representative photoacoustic images demonstrate the accumulation of CAR T cells labeled with ferumoxytol in the tumor + CAR + iron group (green), with a scale bar of 10 mm. Reproduced from^213^ under open access license.

Moreover, nanotechnology enables the design and creation of multifunctional nanoparticles that can deliver CAR T/NK cells while also offering extra features and additional functionalities to enhance their anti‐cancer activity. For instance, researchers have developed nanoparticles that can release immunomodulatory agents or checkpoint inhibitors in combination with CAR cells, generating a synergistic effect that boosts the immune response against cancer.^212^ Additionally, nanomaterials can be engineered to carry imaging agents, allowing for live tracking of CAR T/NK cell distribution and activity within the body. This ability provides valuable insights into the treatment's efficacy and allows for personalized treatment plans based on how each patient responds. A recent study devised a method appropriate for clinical use to label CAR T cells with iron oxide nanoparticles for the noninvasive identification of these labeled T cells using magnetic resonance imaging (MRI), photoacoustic imaging (PAT), and magnetic particle imaging (MPI). Through the utilization of a specialized microfluidics system for T‐cell labeling via mechanoporation, researchers achieved a considerable uptake of nanoparticles in the CAR T cells, while maintaining the cells' ability to proliferate, survive, and function effectively. The combined use of multimodal MRI, PAT, and MPI revealed the migration of the T cells to osteosarcomas and unintended locations in animals that received T cells labeled with iron oxide nanoparticles, whereas T cells were not detectable in animals that received unlabeled cells. This research summaries the successful tagging of CAR T cells with ferumoxytol, opening up possibilities for monitoring CAR T cells in solid tumors^213^ (Figure 6).

The clinical trials of universal CAR‐T cells used antigens derived from allogeneic healthy individuals in order to make the manufacturing process easier. The FDA has recently suspended all clinical trials that involve allogeneic CAR‐T cells because of safety concerns. A secure and simple method of production of CAR‐T cells, therefore, remained an unsolved need. Nanotechnology provides cost‐effective nanocarriers to efficiently and accurately incorporate tumor‐detecting capabilities into the T cells. They are decorated with targeting ligands to lymphocytes, delivering tumor‐specific CAR cargo only to T cells. Following administration, these nanoparticles activate effector cells in large enough numbers to induce tumor regression. In vivo programming with nanoparticles is a new, complex method for simplifying and standardizing the complex manufacturing process of ex vivo CAR‐T cells. Two studies successfully constructed the stable and transient expression of targeting CAR protein in T cells using nanoparticles loaded with CAR‐DNA and CAR‐mRNA, respectively. In both studies, the nano‐delivery system was made up of a cationic polymeric core of poly(β‐amino ester), along with a second‐generation CAR structure targeted to CD19, coated on the outside with PGA, conjugated with an anti‐CD3 antibody. The CD19‐specific CAR gene‐delivered polymer nanoparticles modified T‐cells efficiently and specifically in vivo, bringing about similar anti‐tumor effects to conventionally manufactured CAR T‐cells, with no induction of systemic toxicity.^214^, ^215^ In vivo generation of CAR‐T cells has been investigated using gene delivery vehicles like lentiviruses and AAV, besides polymer nanoparticles.^216^

In vivo CAR‐T‐cell development may face challenges such as reprogramming of senescent and exhausted T cells despite considerable interest. Prolonged exposure to tumor antigens, aging, and intensive treatment of a cancer patient may affect the functionality of immune cells. Therefore, effective strategies are required to be developed before utilizing NPs for in vivo reprogramming of immune cells. Nanocarriers can carry TCR stimulatory signals or pro‐survival cytokines to enhance T‐cell transduction in vivo and boost its survival and proliferation. Research is being conducted on nanoparticles containing IL‐2 and a TGF‐β inhibitor to boost the population of impaired CD8+ T cells and support their survival and activity.^217^

Additionally, nanotechnology can be used to engineer CAR T/NK cells with enhanced functionalities and better abilities to locate and target tumor sites, as well as increased resistance to immunosuppressive factors in the tumor microenvironment. Overall, the integration of nanotechnology into the CAR T/NK cell therapy field shows excessive potential to progress cancer immunotherapy.^218^, ^219^

## 7 FUTURE PERSPECTIVE

Recent improvements in our understanding of the complex immunosuppressive TME have resulted in new approaches and strategies for targeting glioblastoma. Immunotherapy using CAR T/NK cells has emerged as a hopeful treatment strategy against the heterogeneous nature of glioblastoma, and researchers are also exploring combination therapies that target not only the TME but also other aspects of the immune system in the tumor area and the interaction of the immunosuppressive TME and immune system. On the way that CAR T‐cell therapy becomes a standard procedure for treating patients with GBM, there are many issues to be addressed. CAR T‐cell monotherapy has proven ineffective in treating solid tumors due to the numerous immune escape mechanisms utilized by cancer cells in the majority of clinical trials. Combining CAR T/NK cell therapy with checkpoint inhibitors, using small molecule inhibitors that can block key signaling pathways that promote immunosuppression in the TME, and using gene editing techniques to modify the CAR T/NK cells can improve the CAR's efficiency. Therefore, while targeting glioblastoma by CAR T cells is a promising approach, further study is needed to enhance the targeting efficacy of CARs in clinical applications.

Furthermore, the combination of bioengineered CAR T/NK cell therapy with other emerging technologies, such as CRISPR/Cas9 gene editing and artificial intelligence‐driven drug design, holds the potential to optimize treatment outcomes and reduce off‐target effects. Collaborative efforts between researchers, clinicians, and industry partners will be essential in translating these innovative approaches from the bench to the bedside, ultimately improving the prognosis and quality of life for patients with glioblastoma.

In the coming years, we anticipate the development of novel nanocarriers that can encapsulate and protect CAR T/NK cells, thereby prolonging their persistence and enhancing their anti‐tumor activity, and improve the access of cells to brain tumor tissue. Additionally, the integration of advanced imaging modalities with nanotechnology will enable real‐time monitoring of immune cell trafficking and distribution within the brain, leading to personalized treatment strategies for glioblastoma patients.

Nanotechnology has emerged as a powerful tool in enhancing the efficacy and specificity of these cell‐based therapies. As we look ahead, further advancements in nanotechnology are expected to revolutionize the field by enabling targeted delivery of therapeutic agents, enhancing immune cell trafficking to the tumor site, and modulating the immunosuppressive signals within the tumor microenvironment. In conclusion, the junction of bioengineering, nanotechnology, and immunotherapy can revolutionize the landscape of glioblastoma treatment. By harnessing the power of these cutting‐edge technologies, we are on the edge of a new era in precision medicine, where personalized and targeted therapies offer hope for overcoming the challenges of immunosuppression and improving patient outcomes in the fight against this type of cancer.

## AUTHOR CONTRIBUTIONS

**Nasim Dana:** Methodology; writing – original draft. **Arezou Dabiri:** Writing – original draft; methodology. **Majed Bahri Najafi:** Writing – original draft; visualization. **Azadeh Rahimi:** Writing – original draft; data curation; methodology; visualization. **Sayed Mohammad Matin Ishaghi:** Visualization; methodology. **Laleh Shariati:** Writing – original draft. **Minmin Shao:** Writing – original draft. **Assunta Borzacchiello:** Writing – original draft; data curation. **Ilnaz Rahimmanesh:** Conceptualization; validation; writing – review and editing; project administration; funding acquisition; supervision. **Pooyan Makvandi:** Validation; writing – review and editing; supervision.

## CONFLICT OF INTEREST STATEMENT

The authors declared that there was no conflict of interest in this study.

## Data Availability

No new data were created or analyzed in this study, therefore there are no data to share.
